# Time-course transcriptome analyses of spleen in rainbow trout (*Oncorhynchus mykiss*) post-*Flavobacterium psychrophilum* infection

**DOI:** 10.3389/fimmu.2022.965099

**Published:** 2022-08-09

**Authors:** Furong Deng, Di Wang, Thomas P. Loch, Fuguang Chen, Tongyan Lu, Yongsheng Cao, Dan Fan, Shaowu Li

**Affiliations:** ^1^ Department of Aquatic Animal Health, Heilongjiang River Fisheries Research Institute, Chinese Academy of Fishery Sciences, Harbin, China; ^2^ College of Fisheries and Life Science, Shanghai Ocean University, Shanghai, China; ^3^ Key Laboratory of Aquatic Animal Diseases and Immune Technology of Heilongjiang Province, Harbin, China; ^4^ Department of Fisheries and Wildlife, College of Agriculture and Natural Resources, Michigan State University, East Lansing, MI, United States; ^5^ Department of Pathobiology and Diagnostic Investigation, College of Veterinary Medicine, Michigan State University, East Lansing, MI, United States; ^6^ College of Veterinary Medicine, Northeast Agricultural University, Harbin, China

**Keywords:** rainbow trout, spleen, transcriptomic analysis, bacterial coldwater disease, *Flavobacterium psychrophilum*

## Abstract

*Flavobacterium psychrophilum*, the etiological agent of bacterial coldwater disease and rainbow trout fry syndrome, causes considerable losses in salmonid aquaculture globally. Systemic *F. psychrophilum* infections in rainbow trout (*Oncorhynchus mykiss*) lead to a range of clinical signs, including ulcerative lesions in the skin and muscle and splenitis. Previous studies offered an integrative analysis of the skeletal muscle response to *F. psychrophilum* infection in rainbow trout. However, little is known about the molecular mechanism of immune response in the spleen, which is an important immune organ of rainbow trout. Here, we investigated the time-course splenic transcriptome profiles in uninfected rainbow trout (CK) and *F. psychrophilum*–infected rainbow trout at day 3 and day 7 (D3, D7) by RNA-seq analyses. Among the 7,170 differentially expressed genes (DEGs) in the three comparisons (D3 vs. CK, D7 vs. CK, D3 vs. D7), 1,286 DEGs showed consistent upregulation or downregulation at D3 and D7 and were associated with pattern recognition, acute-phase response, complement cascade, chemokine and cytokine signaling, and apoptosis. The Real time quantitative PCR (RT-qPCR) analysis of eight DEGs confirmed the accuracy of the RNA-Sequencing (RNA-seq) data. Our results reflected a general process from pathogen recognition to inflammatory cytokine generation and delineated a putative Toll-like receptor signaling pathway in rainbow trout spleen, following *F. psychrophilum* infection. Taken together, these results provide new insights into the molecular mechanism of the immune response to *F. psychrophilum* infection and are a valuable resource for future research on the prevention and control of bacterial coldwater disease during salmon culture.

## Introduction

The outbreaks of bacterial coldwater disease (BCWD) and rainbow trout fry syndrome continue to cause considerable losses in freshwater salmonid aquaculture around the globe (1). The etiological agent of this disease, *Flavobacterium psychrophilum*, is a Gram-negative rod-shaped bacterium (2) that has been reported throughout Asia, Europe, North America, South America, and Australia (3). Although infections have been reported in other fish species, a wide variety of salmonid species are susceptible during freshwater stages (4). Rainbow trout (*Oncorhynchus mykiss*) infected with *F. psychrophilum* exhibit a range of clinical signs, including ulcerative lesions and necrotic myositis in skeletal muscle (5). Histologically, extensive damage occurs in many tissues, including the liver, spleen, gill, kidney, and intestine wall (6). Among these, spleen and kidney contain the highest levels of *F. psychrophilum*, possibly leading to the suppression of the acquired immune system triggered by this pathogen (7). Likewise, the inflammatory infiltrate of spleen and total splenic ellipsoid increased in rainbow trout post-*F. psychrophilum* infection (8). It was postulated that the spleen size may be an indirect indicator of rainbow trout resistance to *F. psychrophilum* (9), which has led to an increase in studies into the spleen function in teleost fishes (10). However, there is still lack of a comprehensive understanding on the interaction mechanism in rainbow trout spleen after *F. psychrophilum* infection.

Next-generation sequencing technologies have deepened the understanding of immune function and immune-related genes in aquatic animals. Splenic transcriptomes obtained using these technologies have provided important information about the immune response in trout (11, 12). In a previous study, the whole-body gene expression between the genetic lines ARS-Fp-R (resistant) and ARS-Fp-S (susceptible) of rainbow trout was analyzed by high-throughput RNA sequencing (RNA-seq), revealing that 1,884 transcripts, including chemokines, complement components, TNF receptor superfamily members, interleukins, Nucleotide-binding Oligomerization Domain (NOD)-like receptor family members, and genes involved in metabolism and wound healing, exhibited a differential expression between *F. psychrophilum* infection and the control group (13). RNA-Seq has also been applied to uncover the immune response of rainbow trout to several bacterial pathogens like *Lactococcus garvieae* (12) and *Aeromonas salmonicida* (14). Taken together, transcriptome analysis can provide a comprehensive understanding of host immunity and help to uncover the underlying immune mechanisms in aquatic animals in response to pathogen infection.

In the present study, we evaluated the rainbow trout spleen transcriptome in response to *F. psychrophilum* infection using RNA-seq analyses with the aims of: a) providing the transcriptome evidence on the protective effect of acute-phase response (APR), complement systems, Th1-type responses, and caspase-mediated apoptosis in the spleen of rainbow trout against *F. psychrophilum* invasion and b) generating a valuable resource for future research on the prevention and control of BCWD during salmon culture.

## Material and methods

### Bacterial strain isolation and culture

The *F. psychrophilum* isolate CN06, which belongs to ST12 within CC-ST10 (2), originally recovered from the ulcerated muscle of diseased rainbow trout and previously confirmed to be virulent in *in vivo* challenge experiments, was selected for use in the present study. The CN06 strain was grown for 72 h in the TYES broth medium at 15°C, then collected by centrifuging at 6,000 × *g* for 5 min and resuspended to a concentration of 10^8^ Colony-Forming Units (CFU)·ml^−1^ in sterile PBS (pH 7.2). To detect *F. psychrophilum* in the spleen of infected rainbow trout, a PCR assay was conducted following the procedures described by Li et al. (2).

### Bacterial challenge and sampling

Healthy rainbow trout (10–15 g) were obtained from Agrimarine Industrial (Benxi, China), and kept in flow-through tanks for 2 weeks at 14 ± 1°C. During the experiment, 48 rainbow trout (12 fish/m^3^) were fed commercial trout feed twice a day (1.2% of body weight). After verifying the freedom of *F. psychrophilum* infection by bacteriological examination, 48 fish were divided randomly and averaged to four groups: two control groups and two infected groups (12 fish per group). Tricaine methane sulfonate (MS222) was used to anesthetize the fish prior to the challenge and prior to tissue sample collection. The infection was induced experimentally by intramuscular injection in the caudal peduncle with 4 × 10^6^ CFU per gram of fish body weight (LD_50_), whereas fish in the control group were injected with an equivalent volume of phosphate-buffered saline (PBS). Our pre-experiment indicated that rainbow trout showed clinical signs 3 days post-CN06 infection and death occurred 7 days postinfection. Based on the pilot study, the spleen of rainbow trout was euthanized with MS222 and the spleen was collected and frozen immediately in liquid nitrogen at day 3 and day 7 (D3 and D7).

### Messenger RNA (mRNA) library construction and sequencing

Total RNA was extracted using the RNeasy Mini Kit (Qiagen, Austin, TX, USA), following the manufacturer’s instructions. The RNA concentration was measured by Thermo Scientific™ NanoDrop™ 8000 trace ultraviolet-visible spectrophotometer (Thermo Scientific, Wilmington, DE, USA).

A total of 1-µg RNA per sample was used for RNA sample preparations, and sequencing libraries were generated using an NEBNext Ultra™ RNA Library Prep Kit for Illumina (NEB, Ipswich, MA, USA), following the manufacturer’s recommendations, and index codes were added to attribute sequences to each sample. Index-coded samples were clustered on the cBot Cluster Generation System using the TruSeq PE Cluster Kit v4-cBot-HS for Illumina (NEB, USA) according to the manufacturer’s instructions. After cluster generation, library preparations were sequenced on the Illumina platform and double-ended reads were generated.

### RNA-seq read mapping and gene functional annotation

The raw reads were firstly filtered using fastp v 0.20.0 by eliminating adaptor sequences and low-quality reads. The clean reads were mapped to the *O. mykiss* reference genome sequence (genoscope.cns.fr/trout/data/) (15) using Hisat2 (daehwankimlab.github.io/hisat2). Only one mismatch was allowed, and the mapped reads were further analyzed and annotated based on the reference genome data. The Q20, Q30, GC content, and sequence duplication level of the clean data were calculated. The high-quality clean data were used for all the downstream analyses. The mapped reads were annotated by Basic Local Alignment Search Tool (BLAST) searches against the Nr (ncbi.nlm.nih.gov/refseq/release/), Nt (ncbi.nlm.nih.gov/nucleotide/), Pfam (pfam.xfam.org/), Swiss-Prot (expasy.org/resources/uniprotkb-swiss-prot), Cluster of Orthologous Groups of proteins (COG) (ncbi.nlm.nih.gov/research/cog-project/), KO (genome.jp/kegg/), and GO (geneontology.org/) databases. The genes with |log2Ratio| ≥2 and false discovery rate (FDR) ≤0.01 were considered as significantly differentially expressed. The quantification of gene expression levels was estimated by fragments per kilobase of transcript per million fragments mapped (FPKMs). The differential expression analysis of two comparisons (D3 vs. CK and D7 vs. CK) was performed using the DEseq R package. Functional annotation of the DEGs was performed, and the number-annotated DEGs were counted. The GO enrichment analysis was performed using the GOseq R packages, and GO terms with a corrected p-value <0.05 were considered to be enriched. KEGG pathway enrichment was performed using the KOBAS software (16). The TLR signaling pathway was constructed using PathVisio v.3.0.

### Construction of the rainbow trout immune gene library

The classification of rainbow trout immune genes was constructed, following a thorough review of teleost fish immunology (10, 17), and the common carp immune gene library (18) was modified to the rainbow trout immune gene library. The modifications were based on gene information obtained by blasting each sequence to databases, including the non-redundant protein sequences (Nr), Swiss-Prot and Pfam databases. According to both classical immunology and new advances in both fish and mammal immunology, nine categories of immune processes were compiled for this study, including “acute phase reactions”, “pattern recognition”, “antigen processing and regulators”, “complement system”, “inflammatory cytokines and receptors”, “adapters, effectors and signal transducers”, “innate immune cells related”, “T/B cell antigen activation”, and “other genes related to immune response”. Additionally, and because some transcripts expressing respiratory burst activity were revealed, respiratory burst activity was added as an additional category of the immune process. Numerous researched categories of immune genes (detailed in **Table S4**) were applied for the immune process.

### RNA-seq validation by real-time RT-qPCR

To validate the RNA-seq results, eight DEGs including *ccl19*, *casp8*, *infgr1*, *il8*, *c3*, *saa*, *tlr2*, and *tnf10*, which are involved in chemotactic effects, proinflammatory effects, APR, complement cascade, and the Toll-like receptor signaling pathway were further analyzed by RT-qPCR. The RT-qPCR primers are listed in **Table S1**. Gene expression levels were normalized to that of *β-actin*, and data were expressed as the mean ± SE. Differences in means between groups were determined using one-way ANOVA, followed by Bonferroni’s post-test. Data with *p <*0.05 were considered significant. Correlations between the RNA-seq and RT-qPCR data were assessed by multiple linear regression to determine the coefficient of determination (R2) and *p*. All statistical analyses were performed using GraphPad Prism v.9.00 (GraphPad Software, San Diego, CA, CA, USA).

## Results

### Transcriptome sequencing, quality control, and gene alignments

Nine cDNA libraries were constructed from rainbow trout spleen to determine changes in the immune system, following *F. psychrophilum* injection. The raw data generated in each library have been deposited in the NCBI Sequence Read Archive under accession number PRJNA675781. After the quality control of the cDNA libraries, a total of 19,895,398–24,964,841 clean reads were obtained and the percentage of Q30 bases in each sample was more than 92.83% (**Table 1**). All clean reads were mapped to the *O. mykiss* reference genome (NCBI *O. mykiss* Annotation Release 101) (**Table S2**). The normal distribution of gene expression profiling indicated that the three biological replicates had good consistency (**Supplementary Figure S1**). Most reads in each library were mapped to the exon regions of the reference genome (**Supplementary Figure S2**). A total of 8,630 new genes were detected in the three libraries (CK, D3, D7) by RNA-seq analysis, and 74.84% (6,459/8,630) were mapped to the reference genes with homologous sequences in at least one of the multiple databases presented in **Table S3**.

**Table 1:** Characteristics of the reads from nine sample libraries derived from the spleens of *Flavobacterium psychrophilum–*challenged and mock-challenged rainbow trout.

Samples	Clean reads	Clean bases	GC Content	%≥Q30
CK-1	22,353,773	6,673,390,540	49.01%	92.92%
CK-2	24,964,841	7,399,876,374	48.64%	93.41%
CK-3	21,276,059	6,328,664,382	49.00%	93.33%
D3-1	20,660,554	6,158,186,522	48.95%	93.24%
D3-2	21,293,303	6,359,268,762	49.20%	94.07%
D3-3	19,895,398	5,938,294,096	49.52%	93.75%
D7-1	21,905,172	6,529,911,706	48.70%	93.43%
D7-2	21,960,456	6,540,787,578	49.26%	92.98%
D7-3	21,281,242	6,339,892,842	49.05%	92.83%

CK, libraries derived from the spleen tissue of control rainbow trout. D3 and D7, libraries derived from the spleen tissue of F. psychrophilum infected rainbow trout 3 days (D3) and 7 days (D7) postchallenge. Q30, base sequencing error probability <0.1%.

### Analysis of differentially expressed genes

**Figure 1:**
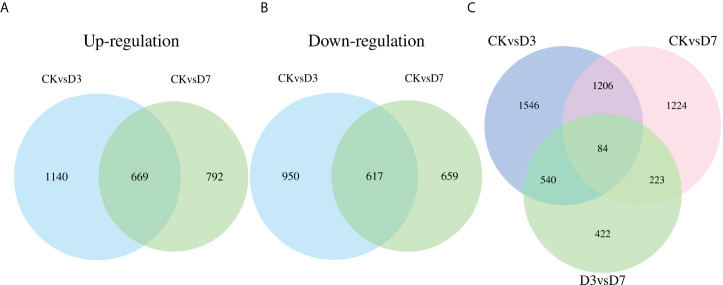
Venn diagrams of differentially expressed genes (DEGs) detected in the spleens of rainbow trout, following *Flavobacterium psychrophilum* and mock challenge infections. **(A, B)** Quantities of up- and downregulated DEGs between groups CK (contro l group) vs. D3 (day 3) and CK vs. D7 (day 7). **(C)** Quantities of DEGs among the three groups.

A total of 7,170 DEGs were identified among the three groups (**Table 2**). Compared with CK, D3 and D7 included 3,376 and 2,737 significant DEGs, respectively. Among them, 1,809 upregulated and 1,567 downregulated genes were identified for CK vs. D3, 1,461 upregulated and 1,276 downregulated genes for CK vs. D7, and 2,601 upregulated and 2,260 downregulated genes for D3 vs. D7 (**Supplementary Figure S3**). Moreover, 1,286 DEGs were common to the CK vs. D3 and CK vs. D7 comparisons, and 84 DEGs were common to all three comparisons (CK vs. D3, CK vs. D7, D3 vs. D7; **Figure 1**).

**Table 2:** The number of annotated differentially expressed genes (DEGs).

DEG set	Total	COG	GO	KEGG	KOG	NR	Pfam	Swiss-prot	EggNOG
CK vs D3	3,279	961	2,295	2,095	2,161	3,266	2,897	2,276	3,106
CK vs D7	2,665	821	1,914	1,724	1,782	2,659	2,355	1,873	2,524
D3 vs D7	1,226	310	903	722	693	1,222	1,065	836	1,145
Total	7,170	2,092	5,112	4,541	4,636	7,147	6,317	4,985	6,775

DEG set: name of differentially expressed gene set; Total: number of differentially expressed genes annotated. COG: Clusters of Orthologous Genes; GO: Gene Ontology database; KEGG: Kyoto Encyclopedia of Genes and Genomes; KOG: Eukaryotic Orthologous Groups of proteins; NR: Non-redundant protein sequences database; Pfam: protein database represented by multiple sequence alignments and hidden Markov models (HMMs); Swiss-prot: protein sequence database with high level of annotation; EggNOG: database of orthologous groups and functional annotation.

### Classification of differentially expressed genes from different comparisons into immune process and immune gene category

**Figure 2:**
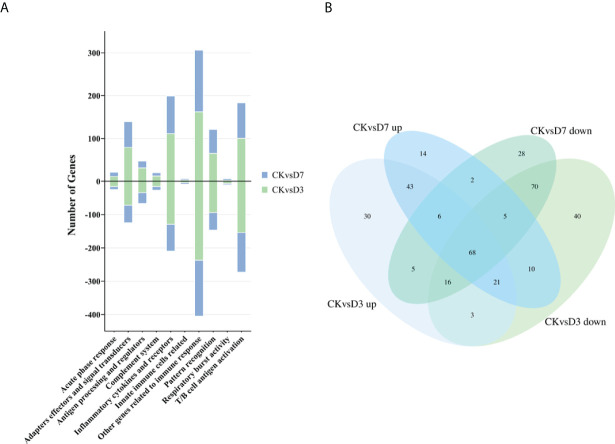
Analysis of the immune-related gene category. **(A)** Number of DEGs belonging to each of the 10 gene categories. **(B)** Venn diagram of the immune gene number of up- or downregulated DEGs in groups CK vs. D3 and CK vs. D7.

We constructed the rainbow trout immune gene libraries including the immune process; gene categories and annotations are presented in the **Table S4**. At the level of immune processing, in group CK vs. D3, most immune-related genes were upregulated in the “adapters effectors and signal transducers,” “antigen processing and regulators,” “inflammatory cytokines and receptors,” “patterns recognition,” “other genes related to immune response,” and “T/B cell antigen activation” (**Figure 2A**, **Table 3** and **S5**). In group CK vs. D7, most immune genes were upregulated in the “adapters effectors and signal transducers”, “inflammatory cytokines and receptors”, “patterns recognition”, “other genes related to immune response,” and “T/B cell antigen activation.” In all groups, “inflammatory cytokines and receptors,” “other genes related to immune response,” and “T/B cell antigen activation” exhibited the greatest DEG number. A Venn diagram was created to demonstrate the relationship between the immune-related DEGs among groups CK vs. D3 and CK vs. D7 (**Figure 2B**); the comparison results of Venn are presented in **Table S6**. Comparison results are synthesized on **Figure 2B**; several DEGs commonly regulated in groups CK vs. D3, and CK vs. D7 within nine immune processes are further depicted (**Table 3**).

**Table 3:** The immune process and immune gene category for overlapping genes in the Venn diagram, DEGs with FC>2 is underlined.

	Immune process	Immune gene category
Common up-regulated genes	acute phase response (4), adapters, effectors and signal transducers (20), antigen processing and regulators (6), complement system (4), inflammatory cytokines and receptors (20), innate immune cells related (2), other genes related to immune response (22), pattern recognition (12), Respiratory Burst Activity (1), T/B cell antigen activation (20),	*cd209, talin, tlr13, cathepsin, fucolectin, macroglobulin, mapk, bcl3, fbxw, cd22, tnip2, hmg, hsp90b, ifn related, Ig light chain, nlrc3, titin, sema, cd40, pkr, hif1a, cdkis, tnfr, egf, fcr, mlph, stat3, cmklr1, nfat, lag3, fam, bap, ildr, immunoglobulin, scavenger receptors, nalp12, maea, nitr, fgf, fgfr, aid, lfa3, ifng, ifngr, nectin, c3ar, cxcl, caspase3, caspase8, ccl, protease, fbxl, mmr, il1r, ifnlr, tal1, zap, il10, hsp30, hsp70, saa, ikbkb, ubl, lysosome, plasminogen, galectin, acrs, mucin, nalp3, topk, fibrinogen, ck2, ifna, traf, tcr, il8, antimicrobial peptides, cbx4, foxp, rxrg, cebp, c1q, c3, c7b, mldl1, nfil3, jak1, itfg, csf, ncf1, ap1, ap3, egfr, hepatic lectin, lrr-containing proteins, ncam, card, oxidoreductase, ube, tgbr, nlrc5, cd166, cxcr, chemotaxin, pias, hsbp, uch, perforin, trap, trim.*
Common down-regulated genes	acute phase response (1), adapters, effectors and signal transducers (10), antigen processing and regulators (8), complement system (1), inflammatory cytokines and receptors (20), innate immune cells related (2), other genes related to immune response (25), pattern recognition (5), Respiratory Burst Activity (2), T/B cell antigen activation (30),	*ubiquitin ligase, slam, il20, mmp, fbh1, bcr related, mapkkk, il17, lag, mapk, nfkb, lrmp, cd22, ifnar, collectin, zcchc, Integrin beta, hdac, vegf, tgfbr, zp, igfn1, cd276, hsp72, irf4, irf5, ifnb, dio1, bcl6b, tnfb, tnfaip, c1q related, igfr, tagap, socs7, uba, tnf10, vtcn, mhc I, bcl11, il21, il6r, fibrinectin, cd3, ap2, cd5, pitp, ttrap, rig, lef1, setbp, il16, cd6, btg, fish-egg lectin, ap4, socs1, sag, bcl11b, caspase2, tbrg, irf2, tlr2, nrros, pik3r, ccr, cklf, rtk, programmed cell death protein, il2r, blnk, ifn related, myeloid cell related, smynd4, bcl7, actinin, nck and related, cdk related, integrin alpha, pI4k, jag, trim31, igdcc3, ildr, tim, palladin, hsp70bp, nalp1, cd97, nfkbi, bcl6, fgfbp, pkc, nkr, integrin related, c-type lectin, bcl11a, wwp, hydrogen peroxidase, selectin, hsp27, cdk, pbox10, pbx, ly86, irf3, vsig.*

Genes marked by solid lines represent CK vs D3, genes marked by dashed lines represent CK vs D7, and genes marked by double solid lines represent CK vs D3 and CK vs D7.

### Gene ontology analysis of differentially expressed genes

**Figure 3:**
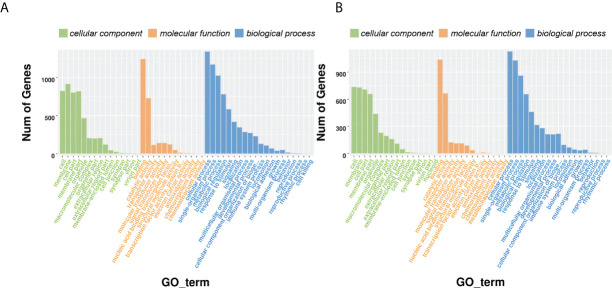
Gene ontology (GO) annotation of the differentially expressed genes. **(A)** CK vs. D3; **(B)** CK vs. D7.

The DEGs were further annotated with terms from the Gene Ontology (GO) database under the three main GO categories: biological process, cellular component, and molecular function. GO terms were assigned to 2,292 and 1,912 DEGs for D3 and D7, respectively (**Figure 3**; **Table S7**). Under cellular component, “cell” and “membrane” were the most enriched terms; under molecular function, “catalytic activity” and “binding” were the most enriched terms; and under biological process, “cellular process”, “single-organism process”, “metabolic process”, “response to stimulus”, “biological regulation”, “signaling”, “localization”, “multicellular organismal process”, “developmental process”, “cellular component organization or biogenesis” and “immune system process” were the most enriched terms. The number of DEGs annotated with the term “immune system process” was higher in CK vs. D3 than it was in CK vs. D7.

To explore the representative GO terms associated with *F. psychrophilum* infection in rainbow trout spleen, the details of the main DEGs that may have important immune functions are listed in **Table 4**. The transcript abundance of cytokine genes *il1β*, *il10*, *il6*, and *il8*; APR gene *saa*; chemokine gene *cc4*, *ccl19a*, *cxcf1b*, and *cxc11*; and complement system genes *c7b*, *c3*, and *c1q* increased in D3 and D7 compared with their abundance in CK, whereas the transcripts associated to the cell membrane including *tlr2*, *itga6*, *itga8*, and the structural molecule activity gene *synemin* decreased.

**Table 4:** Main DEGs annotated with immune-related GO terms.

Gene symbol	Putative homolog protein	CK vs. D3		CK vs. D7	
		Log2	padj	Log2	padj
**Immune system process**					
*saa*	serum amyloid A	10.23	0.00	10.79	0.00
LOC100136024	interleukin-1-beta	9.94	0.00	4.29	0.00
newGene_16692	C-C motif chemokine 4	8.89	0.00	9.64	0.00
*il10*	interleukin 10	9.69	0.00	7.48	0.00
LOC110496949	interleukin 6	8.30	0.00	4.66	0.00
*c7b*	complement component 7b	6.52	0.00	8.16	0.00
LOC110489027	complement C3-like	6.47	0.00	5.87	0.00
LOC110531606	interleukin 8	6.36	0.00	3.36	0.00
LOC110525193	chemokine (C-C motif) ligand 19a	5.63	0.00	4.64	0.00
*cd4-1*	CD4-1 molecule	1.71	0.00	1.16	0.01
LOC100136317	TNF superfamily member 10	-3.03	0.00	-1.48	0.00
*tlr2*	Toll-like receptor 2	-1.81	0.00	-1.46	0.00
**Response to stimulus**					
LOC100136187	cathelicidin antimicrobial peptide	8.76	0.00	10.22	0.00
LOC110489965	atypical chemokine receptor 4-like	6.88	0.00	8.93	0.00
**Membrane part**					
*cd209*	CD209 molecule	5.31	0.00	4.52	0.00
*ifngr1*	interferon gamma receptor 1 precursor	2.29	0.00	1.74	0.00
*synemin*	synemin	-6.39	0.00	-3.83	0.00
LOC110520109	integrin alpha-6-like	-2.49	0.00	-4.28	0.00
**Biological regulation**					
*ITGA8*	integrin alpha-8	-4.64	0.00	-4.15	0.00
**Locomotion**					
LOC110538491	permeability factor 2	8.00	0.00	2.91	0.00
*cxcf1b*	chemokine CXCF1b	5.06	0.00	2.32	0.00
LOC110485791	C-X-C motif chemokine 11	4.25	0.00	2.44	0.00
**Multiorganism process**					
LOC110529346	complement C1q-like	7.42	0.00	6.88	0.00
**Nucleic acid–binding transcription factor activity**					
*fra1*	fos-related antigen 1	6.53	0.00	4.52	0.00
LOC100136256	junB protein	4.09	0.00	2.91	0.00
*fra2*	fos-related antigen 2	3.19	0.00	2.68	0.00

These DEGs were commonly expressed in rainbow trout at D3 and D7 postinfection with F. psychrophilum. CK vs. D3 refers to rainbow trout at 3 days post-infection (D3) compared to uninfected rainbow trout (CK), CK vs D7 refers to rainbow trout at 7 days postinfection (D7) compared to CK, and padj refers to corrected p-Value.

### Pathway enrichment analysis of the differentially expressed genes

**Figure 4:**
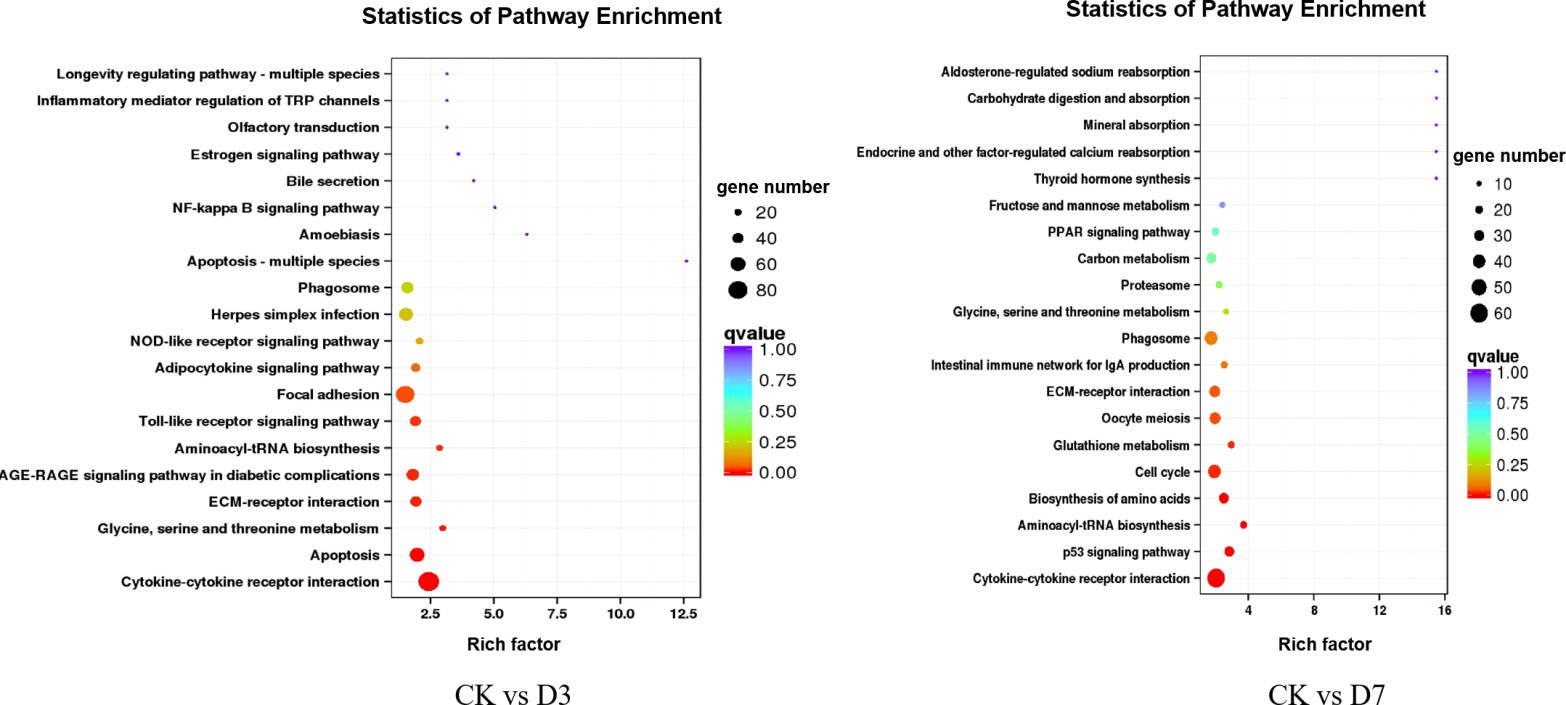
Scatterplot of the KEGG (Kyoto Encyclopedia of Genes and Genomes) pathway enrichment analysis. Rich factor represents the ratio of the number of DEGs and the number of all genes in the pathways.

To further explore the immune-related pathways during *F. psychrophilum* infection in rainbow trout, we performed a KEGG (Kyoto Encyclopedia of Genes and Genomes) pathway enrichment analysis of the DEGs. The DEGs from the CK vs. D3 and CK vs. D7 comparisons were mapped to 161 and 186 KEGG pathways, respectively, whereby 27 and 31 of them were significantly enriched (*p <*0.05). At D3, the DEGs were involved in three immune-related pathways including herpes simplex infection, the NOD-like receptor signaling pathway, and the Toll-like receptor (TLR) signaling pathway. At D7, the DEGs were significantly enriched in the phagosome, oocyte meiosis, cell cycle, and p53 signaling pathways (**Figure 4**).

**Figure 5:**
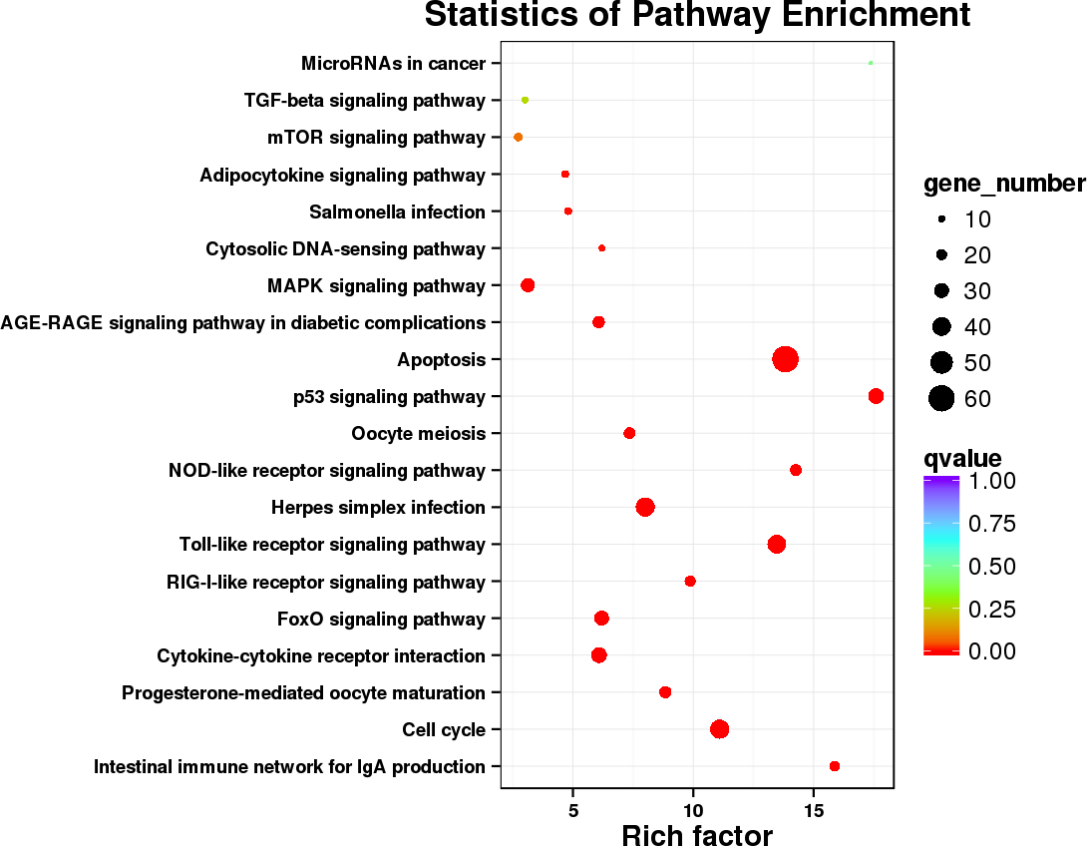
Scatterplot of the KEGG pathways involved in the immune system and cell growth and death. Rich factor represents the ratio of the number of DEGs and the number of all genes in the pathways.

Significantly enriched pathways were mainly classified as immune system and cell growth and death. Thereby, DEGs annotated in these two subcategories were selected and re-enriched for the more explicit functions, as shown in **Figure 5**. A total of 1,286 DEGs were common to CK vs. D3 and CK vs. D7, and 210 genes in these two subcategories were annotated to 18 KEGG pathways. Among them, apoptosis, Toll-like receptor signaling pathway, cell cycle and p53 signaling pathway were significantly enriched distinguished to other pathways, both in the gene number and the rich factor.

**Figure 6:**
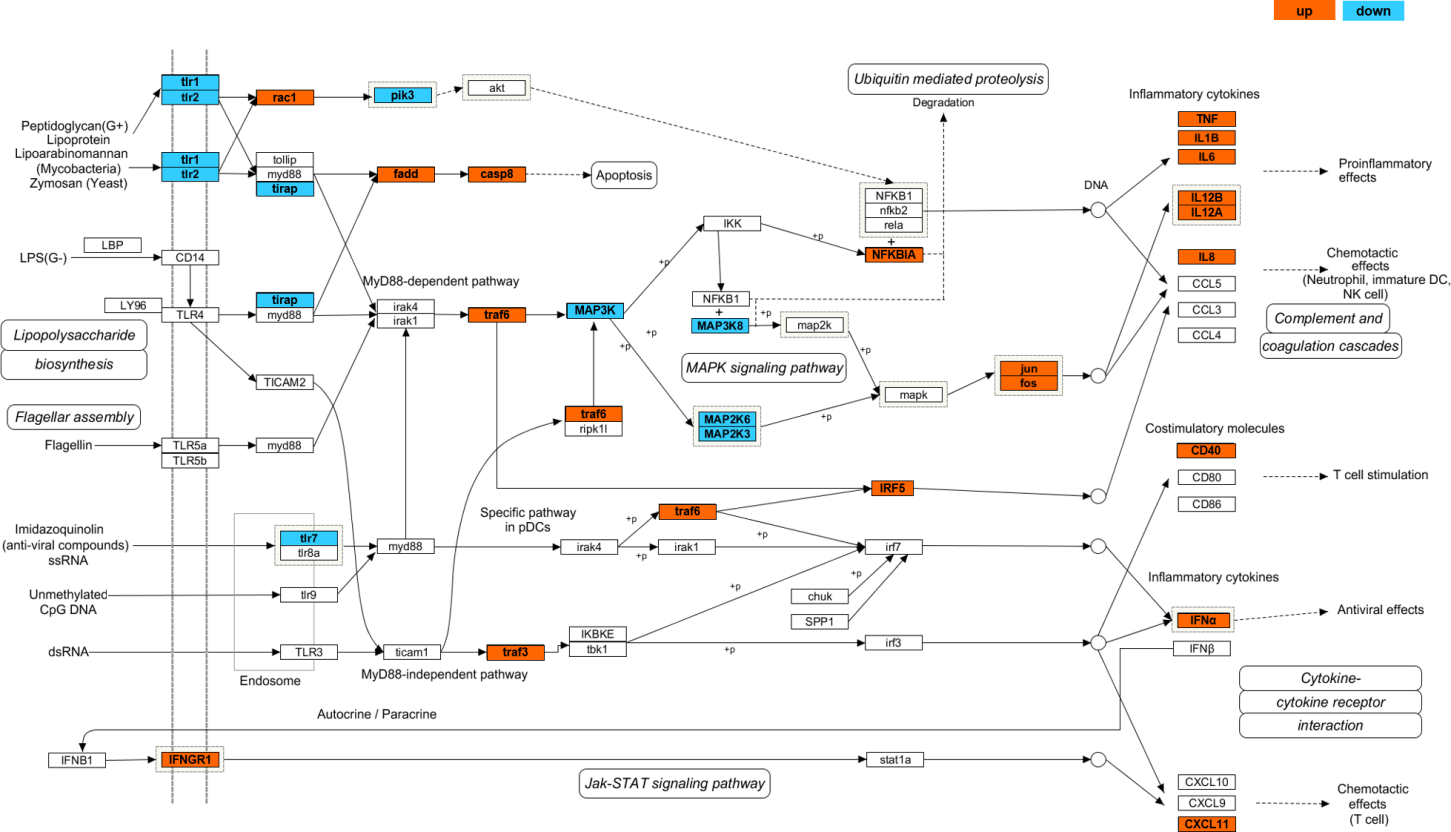
Predicted integrated Toll-like receptor signaling pathway in rainbow trout spleen. The pathway was constructed based on the pathway in *Danio rerio* (zebrafish) using PathVisio v3. Blue and red indicate a decrease and increase in any of component of the pathways, respectively, which contains D3 and D7 time points postinfection.

The immune-related DEGs in the TLR signaling pathway mainly contained cytokines, chemokines, and APR genes, such as APR *saa*; inflammatory cytokines *il8*, *il1β*, and *il6*; interferon receptor *ifngr1*; apoptosis pathway genes *casp8* and *casp3*; ubiquitin-mediated proteolysis *nfkbie* and *nfkbia*, and oncogenic transcription factors *jun* and *fos*, which were upregulated expression at D3 and D7 (**Figure 6**). In contrast, TLRs such as *tlr7*, *tlr9*, *tlr2*, and *tril* showed downregulated expression at D3 and D7.

### Validation of selected differentially expressed genes by RT-qPCR analysis

**Figure 7:**
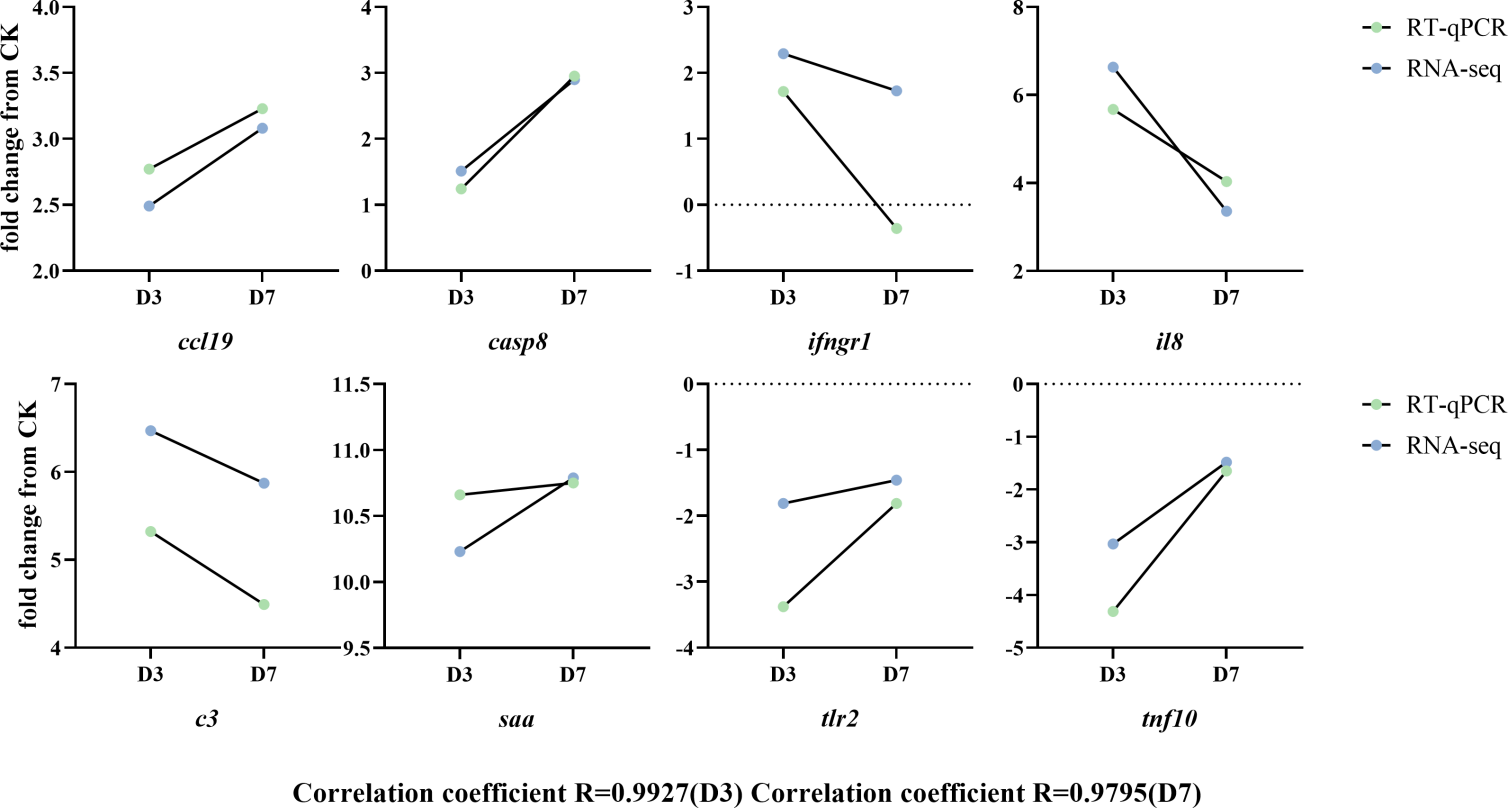
Validation of selected differentially expressed genes by RT-qPCR. Fold changes in rainbow trout at 3 and 7 days postinfection (D3 and D7) with *F. psychrophilum* are expressed as the ratios of gene expression at D3 and D7 to gene expression in the CK as normalized with the *β-actin* gene*. ccl19*, C-C chemokine ligand 19; *c3*, complement C3; *casp8*, caspase 8; *saa*, serum amyloid A; *ifngr1*, interferon gamma receptor 1 precursor; *tlr2*, Toll-like receptor 2; *il8*, interleukin 8/permeability factor 2; *tnf10*, TNF superfamily member 10.

To validate the RNA-seq analyses, eight immune-related DEGs containing both up- and down-regulated genes were chosen for RT-qPCR analysis (**Figure 7**). The gene expression fold changes at D3 and D7 measured by RT-qPCR were highly correlated with those obtained by RNA-seq analysis. The significant coefficients between RT-qPCR and RNA-seq for the DEGs in D3 and D7 groups were 0.99 and 0.98, respectively.

## Discussion

Previous studies have described that the spleen size is an indirect indicator of immunological status in histologically (9). The mechanisms of immune response to *F. psychrophilum* in spleen are the key events which are vital matters to the interactions occurring in rainbow trout post bacterial infection. But until this study, the transcriptional response in the spleen of rainbow trout against *F. psychrophilum* infection was not fully understood. Herein and for the first time, attempts to elucidate this mechanism by transcriptome analysis have been performed to explore immunological insensitivity or susceptibility factors to *F. psychrophilum* infections in the present study. And our results have novel findings and identify the interactions between *F. psychrophilum* and its susceptible host rainbow trout.

### Differentially expressed genes related to the pattern recognition receptors

Pattern recognition receptors (PRRs), including TLRs, C-type lectin receptors (CLRs), scavenger receptors (SRs), and nucleotide oligomerization domain–like receptors (NLRs), are critical for bony fish to develop a higher specialization in innate immunity after recognizing the pathogens. TLRs play an integrated role as a first line of defense against invading pathogens, where they contribute to the inflammation, immune cell regulation, survival, and proliferation of leukocytes. Here, we found that *tlr2* expression was downregulated at D3 and D7, suggesting that it may play a negative regulatory role in rainbow trout immune defense, following *F. psychrophilum* infection. Different to our results, Tang et al. provided the evidence that TLRs in sturgeon showed upregulated expression in head–kidney primary leukoocytes, following LPS and polyI:C stimulation (19). In epithelial or mucosal barriers, *tlr2* transcripts in rainbow trout skin were upregulated both at 4 h and 2 d post-immersion vaccination, suggesting that *tlr2* was in contact with *F. psychrophilum* to initiate the subsequent immune response (7). Moreover, *tlr7* expression was unchanged at D3 but downregulated at D7, consistent with previous studies (21). Most of the CLRs induce activating or regulatory signal cascades in response to distinct pathogen- or self-derived components. CD209 is shown to be an essential surface factor of the immune defense of rainbow trout, by recognizing high-mannose-containing glycoproteins on pathogens’ outer surfaces (14), and TLR13α1 is responsible for CD209 regulation (24). In the present study, we observed a dramatic increase of the expression level of *cd209* and *tlr13* in rainbow trout spleen at D3 and D7 post-*F. psychrophilum* infection (**Table S5**). Consistent with the findings of Long et al. (14), the *tlr13α1* and *cd209* genes were both transcriptionally upregulated in the spleens of fish experimentally challenged with another Gram-negative bacterial pathogen of salmonids, *A. salmonicida.* Of the CLRs, upregulated expression was detected in *collectin*, *fish-egg lectin*, and *selectin*, which could potentially be specific to various pathogens in Japanese eel (*Anguilla japonica*), European eel (*Anguilla Anguilla*), and zebrafish (29). The *scavenger receptor*, also presented in our analysis (**Table S5**), was dramatically upregulated in rainbow trout spleen post-*F. psychrophilum* infection at D7. Less studies were reported about SRs in rainbow trout by far. Qiu et al. (20) demonstrated that two variants of rainbow trout SRs with a collagenous structure were identified and found to bind to Gram-negative and -positive bacteria. Our results indicated that SRs may take part in innate immune regulation in rainbow trout against *F. psychrophilum* infection.

### Differentially expressed genes in Toll-like receptor pathway

To better understand the function of TLRs in the immune response of rainbow trout spleen against *F. pyschrophilum* challenge, we focused on several transcripts involved in the TLR signaling pathway in this research. The TLR signaling pathway both initiates the overactive innate response and activates antigen-producing cells, thus participating in adaptive immune response in fish. Toll-interacting protein (Tollip) can inhibit TLR-mediated cellular responses by suppressing phosphorylation and the kinase activity of IRAK1 (22). Our results showed that the *irak1* and *irak4* expression levels were unchanged in rainbow trout infected with *F. psychrophilum*, but one of the components of *tollip* and *traf6* were upregulated at D3 and D7. TRAF6 could mediate downstream signaling cascades in the immune response, but its function in inhibiting the activity of IRAK1 requires further investigation. TRIL (functional component of TLR4) and AP-1 (complex within jun and fos), which is the signal transduction protein in the endpoint of the NF-κB pathway, genes were down- and upregulated postinfection, respectively, which may explain the negative regulation of TRIL in the TLR signaling cascade. Coincidentally, Sepulcre et al. (23) have proven that LPS signaled *via* a TLR4- and MyD88-independent manner in fish, while the zebrafish TLR4 orthologs negatively regulated the MyD88-dependent signaling pathway. Srivastava et al. (25) demonstrated that the TLR4-TRIF-NFκB axis promotes the rapid secretion of anti-inflammatory cytokines. In addition, *ifn-γ* (*ifng*) in the Toll-like pathway not only has anti-viral activity (26) but also assists in the autophagy of intracellular bacteria (27). Our analysis (**Table 4**) showed that both *ifng* and *ifngr* showed upregulated expression, following infection with *F. psychrophilum*, suggesting that *ifng* may also play an important role in the immune response of infected rainbow trout. The importance of the TLR signaling pathway–mediated immune response in protection against *F. psychrophilum* infection needs further investigation.

### Differentially expressed genes related to the cytokines

Cytokines, including interferons (IFNs), interleukins (ILs), tumor necrosis factors (TNFs), colony-stimulating factors, and chemokines, could stimulate T and B cells and antigen-presenting cells, mediating the communication between innate and acquired immune in fish. In rainbow trout, higher cytokine abundance in the spleen correlated strongly with lower *F. psychrophilum* loads in challenged fish (28). In the present study, the transcript levels of *il1β1*, *il1β2*, *il6*, and *il8* continuously increased in the spleen of fish postexposure to *F. psychrophilum* (at D3 and D7), whereas *tnf-α* was upregulated at D3 and downregulated slightly at D7 (**Figure 7**, **Table S5**). Similar results were reported in rainbow trout infected with *F. psychrophilum* by Kutyrev et al. (30), who found that pro-inflammatory cytokine gene transcripts including *il1β1*, *il1β2*, and *tnf-α* in the spleen were upregulated till 72 h. Hou and Xin et al. (31) evaluated the transcript profiling between symptomatic and asymptomatic trout after *Vibrio anguillarum* injection and confirmed that cytokines in the spleen including *il1β*1, *il1β*2, *il8*, and *tnf-α* were dramatically increased (FC>8) in symptomatic trout. Among atypical chemokine receptors, *ccl*, *cxcl*, and *ck2* transcripts showed a consistent upregulation after *F. psychrophilum* infection, similar to previous studies (32, 33). The Th1-type cytokine *ifn-γ* (34), which is induced by *il12* in natural killer cells and T cells, was significantly upregulated (FC>4) at D3, but showed no remarkable change at D7. Some interferon regulatory factors (*irf1*, *irf3*, *irf4b*, *irf5*, *irf6*, *irf8*) continued to show upregulation during the later stages of infection (day 6 to day 10) in virus-infected fish spleens (35). Based on our data, *irf1*, *irf7*, and *irf8* were upregulated at D3. *irf2*, *irf3*, and *irf4* were downregulated both at D3 and D7, while *irf5* was downregulated at D7.

### Differentially expressed genes related to complement systems

The activation of a complement system helps the adaptive immune system to more effectively eliminate the foreign pathogens. Specific complement components were found to be regulated under different rearing conditions (36). Marancik et al. (13) found that C3- and C1q-like proteins were upregulated in rainbow trout, following an experimental challenge with *F. psychrophilum*. In the present study, the complement cascade response genes, such as *c7b*, *c3*, *c3ar*, and *c1q-like*, were significantly upregulated at D3 and D7 (FC>6). C1q and C3 regulate classical and alternative complement pathways, respectively (37). Our results indicated that complement systems were activated and may constitute an effective response in rainbow trout post-*F. psychrophilum* infection (13).

### Differentially expressed genes related to antigen processing and presentation

In fish, antigen presentation appears to take place locally where the antigen is present. The major histocompatibility complex (MHC II) is involved in the presentation of exogenous antigens by specialists such as macrophages, dendritic cells, and B lymphocytes in fish. Th lymphocytes were activated and secrete cytokines to modulate the adaptive immune response after recognizing this MHC II antigen complex (38). Jirillo et al. (39) demonstrated that IFN-γ and MHC II β chain also enhance MØ respiratory burst, thus enabling the clearance of antigens. It was shown that the ability of rainbow trout gut to mount a T-cell-dependent delayed-type hypersensitivity response based on the release of MIF and of an IFN-γ-like factor (39). *mif* and *mhc-iiβ* (**Table S5**) were upregulated in rainbow trout after the *F. psychrophilum* challenge, which inferred the efficient cell–cell interactions within the immune system. Therefore, these memory cells can react rapidly to a second antigen encounter and are essential for successful vaccination. Here, we observed that MIF was upregulated at D3 and the MHC II β chain was upregulated at D7. Interestingly, it has been proven that the MHC II β chain could induce the expression of *tnf-α* and *il6* in fish and regulate and coordinate the interaction between cytokines and their receptors (40, 41). It may suggest that the change of MIF and the MHC II b chain on the surface of antigen-presenting cells could stimulate the secretion of cytokines and activation of Th1-type responses in spleen of rainbow trout post F. psychrophilum infection.

### Differentially expressed genes related to other immune gene pathways

Serum amyloid A (SAA) is considered a major protein in the APR and is an effector of innate immunity in all vertebrates. We found that *saa* was upregulated (FC >10) at D3 and D7, indicating the activation of APR after infection, similar to the previous studies (42, 43). Fibrinogen and macroglobulin are involved in the early innate immune system to rapidly isolate and kill invading pathogens (44). As shown in **Table 4**, both *macroglobulin* and *fibrinogen* were upregulated at two time points after infection, suggesting that the pathways activated by the APR were essential antibacterial mechanisms for rainbow trout infected with *F. psychrophilum*. Cell apoptosis could eliminate microbes and cellular remains and remove inflammatory exudates in an innate immune response. Zeng et al. (45) showed that the apoptosis executioner *casp3* and apoptosis initiator *casp8* were upregulated in rainbow trout after *A. salmonicida* and *V. anguillarum* challenges, which regulated intrinsic and extrinsic apoptosis, respectively. In this study, *casp8* and *casp3* were upregulated at D3 and D7, while both *casp7* and *casp14* were significantly upregulated at D3 (FC>7). These results were in accordance with the reports of Aravena (5), which indicated that the *F. psychrophilum* challenge could induce an increase of caspase-cleaved proteins in the skeletal muscle of rainbow trout. Fu et al. (46) showed that *casp7* was involved in apoptosis in puffer fish after a bacterial infection and was dramatically upregulated, consistent with our results.

## Conclusion

In the present study, a splenic transcriptome analysis for *F. psychrophilum–*infected rainbow trout was performed to explore the molecular immune mechanisms for the first time. The DEGs that were induced by *F. psychrophilum* infection and might be related to immune responses were identified, annotated, and analyzed. PRRs, complement molecules, inflammatory cytokines, chemokines, major histocompatibility complexes, and other immune-related genes might participate in the immune responses of rainbow trout to *F. psychrophilum*. In conclusion, this work provides a better understanding of the immune system and the defense mechanisms of rainbow trout against *F. psychrophilum*.

## Data availability statement

The datasets presented in this study can be found in online repositories. The names of the repository/repositories and accession number(s) can be found in the article/**Supplementary Material**.

## Ethics statement

The animal study was reviewed and approved by The Committee of the Ethics on Animal Care and Experiments at Heilongjiang River Fisheries Research Institute of Chinese Academy of Fishery Sciences.

## Author contributions

SL and TYL designed the study. FD, DW, and YC carried out the experiments. FD and DW performed data interpretation and drafted the manuscript. FC and DF performed RT-qPCR analysis. TPL and SL provided a critical editing of the manuscript. All authors contributed to the article and approved the submitted version.

## Funding

This work was supported by Central Public-interest Scientific Institution Basal Research Fund, HRFRI (NO. HSY202001M) and Central Public-interest Scientific Institution Basal Research Fund, CAFS (NO. 2020TD43).

## Acknowledgments

We thank BioMarker Technologies Corporation for providing RNA-seq service and BMKcloud Platform for data analysis.

## Conflict of interest

The authors declare that the research was conducted in the absence of any commercial or financial relationships that could be construed as a potential conflict of interest.

## Publisher’s note

All claims expressed in this article are solely those of the authors and do not necessarily represent those of their affiliated organizations, or those of the publisher, the editors and the reviewers. Any product that may be evaluated in this article, or claim that may be made by its manufacturer, is not guaranteed or endorsed by the publisher.
