# Supplementary figures and images for: Time-course transcriptome analyses of spleen in rainbow trout (Oncorhynchus mykiss) post-Flavobacterium psychrophilum infection
Source: Front Immunol. 2022 Aug 9;13:965099. doi: 10.3389/fimmu.2022.965099 (PMC9396386; doi:10.3389/fimmu.2022.965099)

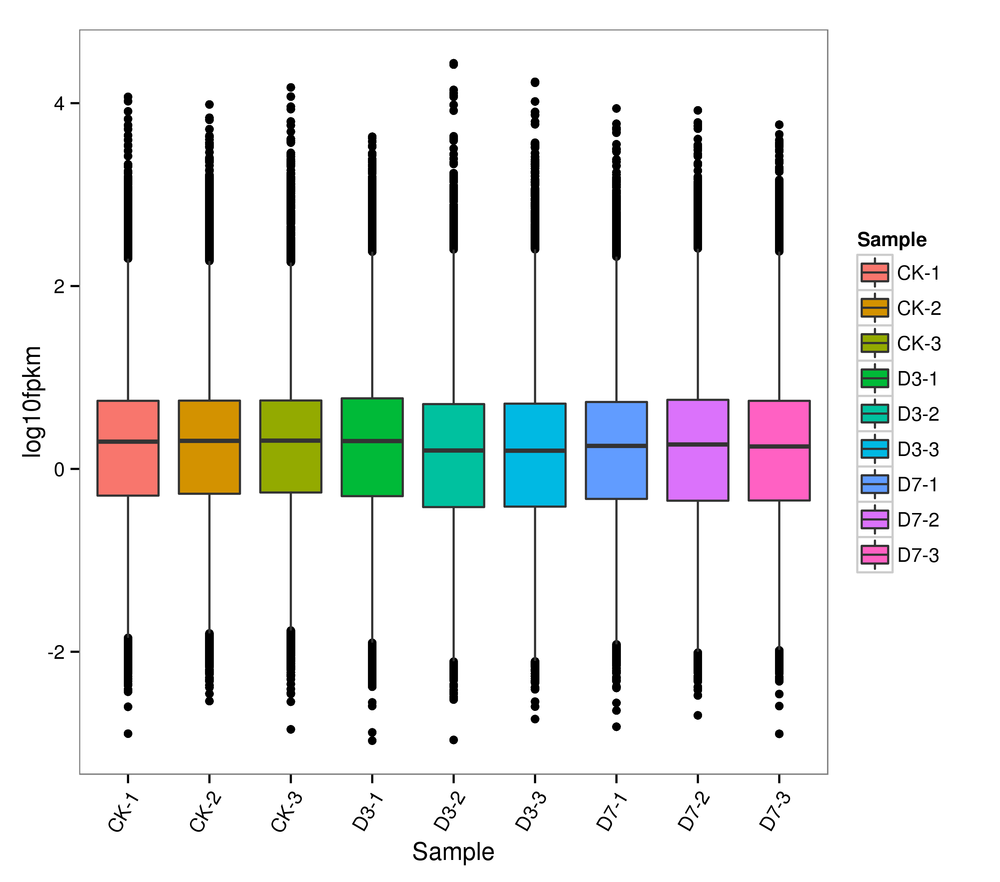

Supplement: Supplementary file 1 [file Image_1.tif]

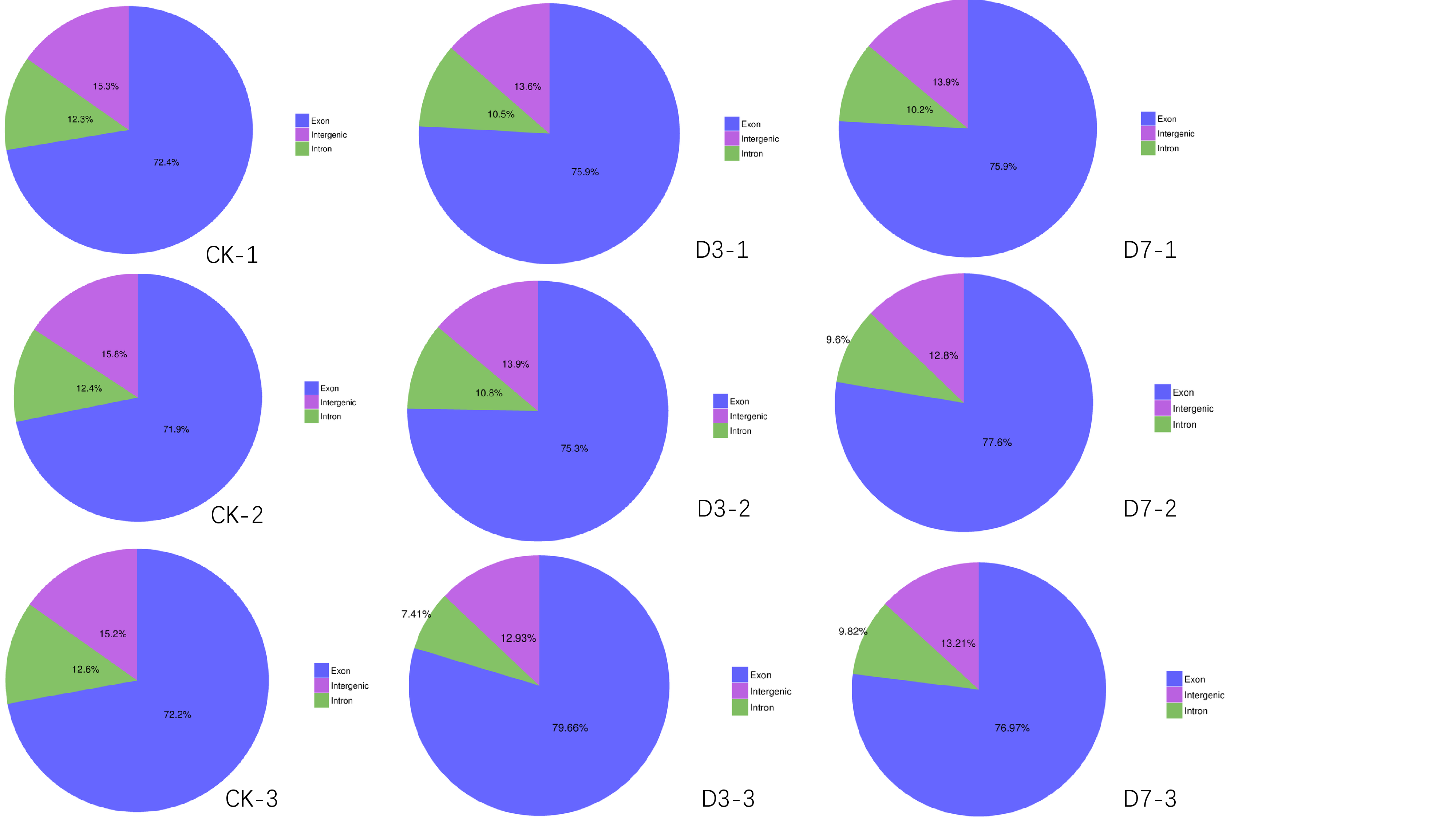

Supplement: Supplementary file 2 [file Image_2.tif]

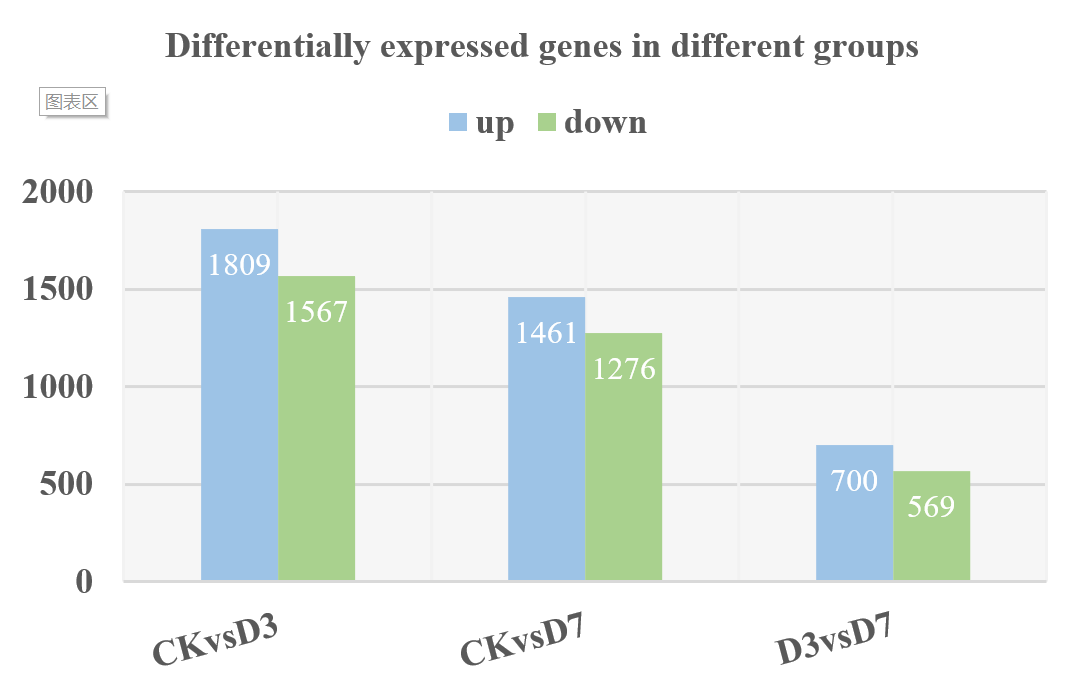

Supplement: Supplementary file 3 [file Image_3.tif]
